# Optical Fiber Temperature and Torsion Sensor Based on Lyot-Sagnac Interferometer

**DOI:** 10.3390/s16101774

**Published:** 2016-10-24

**Authors:** Li-Yang Shao, Xinpu Zhang, Haijun He, Zhiyong Zhang, Xihua Zou, Bin Luo, Wei Pan, Lianshan Yan

**Affiliations:** Center for Information Photonics & Communications, School of Information Science and Technology, Southwest Jiaotong University, Chengdu 611756, China; haijunhe@my.swjtu.edu.cn (H.H.); zhiyongzhang@home.swjtu.edu.cn (Z.Z.); zouxihua@home.swjtu.edu.cn (X.Z.); bluo@home.swjtu.edu.cn (B.L.); wpan@home.swjtu.edu.cn (W.P.); lsyan@home.swjtu.edu.cn (L.Y.)

**Keywords:** fiber sensor, temperature and torsion sensor, Lyot-Sagnac interferometer, high-birefringence fiber

## Abstract

An optical fiber temperature and torsion sensor has been proposed by employing the Lyot-Sagnac interferometer, which was composed by inserting two sections of high-birefringence (HiBi) fiber into the Sagnac loop. The two inserted sections of HiBi fiber have different functions; while one section acts as the temperature sensitive region, the other can be used as reference fiber. The temperature and twist sensor based on the proposed interferometer structure have been experimentally demonstrated. The experimental results show that the envelope of the output spectrum will shift with the temperature evolution. The temperature sensitivity is calculated to be −17.99 nm/°C, which is enlarged over 12 times compared to that of the single Sagnac interferometer. Additionally, the fringe visibility of the spectrum will change due to the fiber twist, and the test results reveal that the fringe visibility and twist angle perfectly conform to a Sine relationship over a 360° twist angle. Consequently, simultaneous torsion and temperature measurement could be realized by detecting the envelope shift and fringe visibility of the spectrum.

## 1. Introduction

Fiber sensors based on a Sagnac interferometer have been widely used in parameter monitoring due to their potential applications and their distinctive advantages over traditional sensors, such as immunity to electromagnetic radiation, resistance to chemical corrosion, miniature size, high accuracy, and remote operation [1,2]. Normally, when a section of high-birefringence (HiBi) Fiber is inserted in the Sagnac loop, this will introduce optical path difference between two counter-propagating waves and cause an interference spectrum, which can be used as investigation signal for sensing [3,4]. Recently, the applications of fiber Sagnac interferometer-based sensors are the subject of considerable research, such as temperature sensors [5,6], pressure sensors [7,8], strain sensors [9,10], and biochemical sensors [11,12]. Among them, temperature sensors based on Sagnac interferometers with conventional HiBi fiber have been demonstrated and used in high-sensitivity temperature sensing (about 1.46 nm/°C) [13]. Especially, Qian et al. proposed an alcohol-filled HiBi-PCF (photonic crystal fiber) Sagnac sensor to improve the sensitivity up to 6.6 nm/°C [14]. Dong proposed a sensitivity-enhanced temperature sensor based on the cladding mode interference by splicing a mode mis-matched dispersion compensation fiber between single mode fibers [15]. Yan et al. demonstrated highly-sensitive temperature and strain sensors based on an all-fiber Lyot filter structure, which is formed by concatenating two 45°-tilted fiber gratings with a polarization-maintaining fiber cavity [16,17]. However, the fabrication of this sensor requires complicated and time-consuming fabricating procedures, which increase the cost and complexity of fabrication and operation. Although this method can provide high-sensitivity measurement, the sensor fabrication procedures are complicated, which restricts the practical applications of this sensor. Besides, torsion sensors are an important application of fiber Sagnac interferometers [18]. However, the Sagnac interferometer based on low-birefringence photonic crystal fiber is temperature-insensitive, making it difficult to obtain real-time temperature variation and realize simultaneous measurement of temperature and torsion.

In this paper, we present a Lyot-Sagnac interferometer by inserting two sections of high-birefringence fibers and two polarization controllers (PCs) into the fiber loop, and experimentally demonstrate its high sensitivity temperature and twist-sensing capability. The experimental results show that the proposed sensor has high sensitivity to temperature, and a 17.99 nm/°C temperature sensitivity is obtained. Furthermore, we calibrated and tested the twist responses of the proposed sensor, and the test results reveal that the fringe visibility and twist angle perfectly conform to Sine relationship. Therefore, the twist angle can be detected and evaluated by measuring the evolution of the fringe visibility.

## 2. Operation Principle

**Figure 1:**
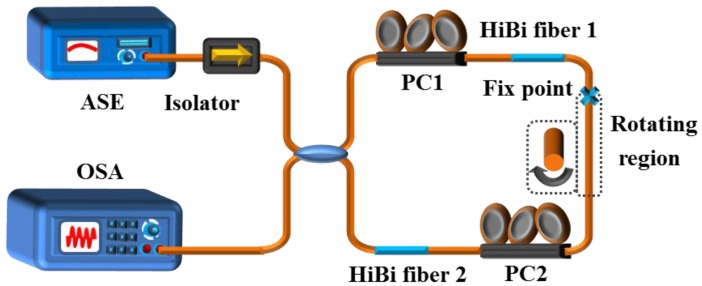
Experimental setup of the Lyot-Sagnac interferometer. ASE: Amplified spontaneous emission; HiBi: High-birefringence; OSA: Optical spectrum analyzer; PC: Polarization controller.

The schematic diagram of the proposed Lyot-Sagnac interferometer is shown in Figure 1. The light source used in the experiment is a broadband light source, and an optical spectrum analyzer (OSA) was employed to measure the interference spectra. The Sagnac interferometer consists of a conventional 3-dB single-mode fiber (SMF) coupler and two inserted sections of HiBi fiber within the fiber loop. As shown in Figure 1, the 3 dB coupler splits the input signal equally into two signals. The two signals counter-propagate through the fiber loop before they interfere again at the coupler. With a broadband light source at the input, the output transmission spectrum is approximately a periodic function of the wavelength. 

The two inserted sections of HiBi fiber have different functions; while one section is used as the temperature sensitive element, the other can act as reference fiber. The temperature perturbation-induced birefringence change of HiBi fiber 1 results in a phase difference between the two signals and then causes a shift of the output interference spectrum. Temperature variation can be determined by measuring the wavelength shift of the interference spectrum. To verify the torsion characteristics of the Sagnac interferometers, we induced a torsion device on the single mode fiber between the two sections of HiBi fiber. One end of the SMF was fixed firmly, and the other end was twisted by the torsion device.

As mentioned above, the transmission spectrum of the Lyot-Sagnac interferometer can be given by [19]

(1) $$T={\left[\cos\left(\frac{\varphi_{1}+\varphi_{2}}{2}\right)\sin(\theta_{1}+\theta_{3})\cos\theta_{2}+\cos\left(\frac{\varphi_{1}-\varphi_{2}}{2}\right)\cos(\theta_{1}+\theta_{3})\sin\theta_{2}\right]}^{2}$$

where $\varphi_{1}=2\pi BL_{1}/\lambda$ and $\varphi_{2}=2\pi BL_{2}/\lambda$ are the phase differences between the two orthogonal modes caused by HiBi fiber 1 (length *L*_1_) and HiBi fiber 2 (length *L*_2_), respectively. $\theta_{1}$ and $\theta_{3}$ are the angles between the polarization directions of counter propagating beams and the corresponding fast axes of the two sections of HiBi fiber. $\theta_{2}$ is the angle between the fast axes of the two sections of HiBi fiber. In the Sagnac interferometer, HiBi fiber 1 functions as the temperature sensing fiber; when HiBi fiber 2 is isolated to external temperature perturbations, $\varphi_{1}$ can be expressed as

(2) $$\varphi_{1}=\varphi_{10}+\Delta \varphi_{10}=2\pi \left(B+K_{t}\Delta T\right)L_{1}/\lambda$$

where *K_t_* and ∆*T* are the birefringence-temperature coefficient and the variations of temperature, respectively. 

Additionally, $\theta_{2}$ (the angle between the fast axes of the two sections of HiBi fiber) can be modulated by torsion, and the $\Delta \theta_{2}$ can be given by [18]

(3) $$\Delta \theta_{2}=g\phi$$

where ϕ and g are the twist angle and a constant of SMF, respectively, and *g* depends on the photoelastic coefficients of the material; for a standard SMF, *g* = 0.08. Therefore, Equation (1) can be written as

(4) $$T={\left[\cos\left(\frac{\varphi_{1}+\Delta \varphi_{1}+\varphi_{2}}{2}\right)\sin(\theta_{1}+\theta_{3})\cos(\theta_{2}+g\phi )+\cos\left(\frac{\varphi_{1}+\Delta \varphi_{1}-\varphi_{2}}{2}\right)\cos(\theta_{1}+\theta_{3})\sin(\theta_{2}+g\phi )\right]}^{2}$$

where the change of $\Delta \varphi_{1}$ and $\Delta \theta_{2}$ are completely independent of one another, $\Delta \varphi_{1}$ is caused by temperature perturbations, and $\Delta \theta_{2}$ is determined only by torsion, so this interferometer can be used for simultaneous temperature and torsion measurement.

## 3. Experiment and Results

**Figure 2:**
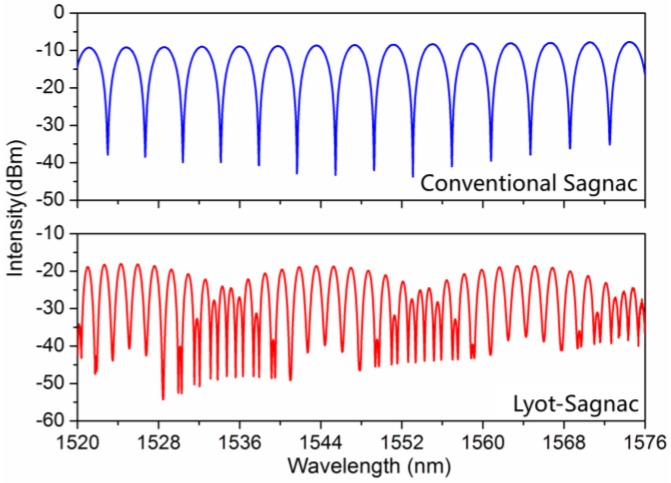
Spectra of the conventional Sagnac and Lyot-Sagnac interferometers.

In the experiment, a broadband amplified spontaneous emission (ASE) source with 60 nm wavelength range was used as a light source. The transmission spectrum of the Lyot-Sagnac interferometer was measured by an optical spectrum analyzer with a wavelength resolution of 0.02 nm. The HiBi fiber used here was Panda fiber (PM1550 125-18/250) fabricated by Yangtze Optical Fiber and Cable Company Ltd.; the phase modal birefringence B is about 6.9 × 10^−4^. HiBi fibers (2.2 m and 2 m) were inserted into the fiber loop and labeled as HiBi fiber 1 and HiBi fiber 2, respectively. Figure 2 illustrates the spectra of the conventional Sagnac and Lyot-Sagnac interferometers at the 1550 nm wavelength band (at 20 °C, without torsion). From Figure 2, we can clearly observe that the Lyot-Sagnac interferometer has a more dense free spectral range and sharper resonance peaks than the conventional Sagnac interferometer, which is beneficial for the sensor resolution. The extinction ratio of the Lyot-Sagnac interferometer between the transmission maxima and the transmission minima is about 26 dB around 1550 nm. The spacing between two adjacent transmission minima is 1.73 nm at 1550 nm.

**Figure 3:**
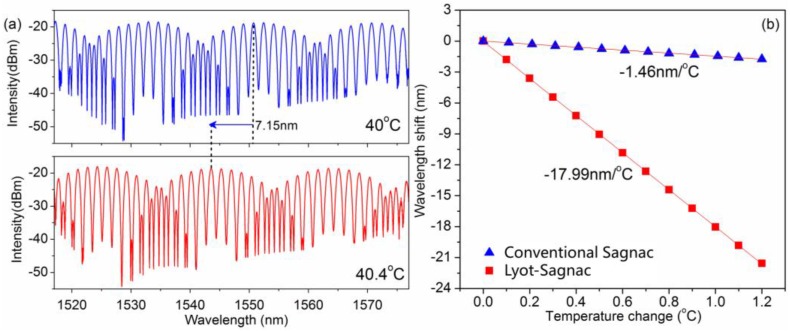
(**a**) Spectra of Lyot-Sagnac interferometer at 40.0 °C and 40.4 °C; (**b**) Temperature responses of the Lyot-Sagnac interferometer.

During the individual temperature sensing process, HiBi fiber 1 was put into a programmable tubular furnace for temperature testing; in order to realize easy measurement, HiBi fiber 1 (temperature sensing fiber) was coiled into a small circle to put into the programmable tubular furnace, and a thermocouple was used for standard temperature calibration near HiBi fiber 1. We characterized the temperature performance by tracking a peak of the transmission spectrum from the Sagnac interferometer. The temperature range was increased from 40 to 41 °C with a step size of 0.1 °C. Figure 3a presents the transmission spectra of this fiber Lyot-Sagnac interferometer versus temperature variations at 40 and 40.4 °C. As the temperature increases, there are obvious spectrum shifts. As shown in Figure 3a, the spectrum of the Lyot-Sagnac interferometer has significant blue-shifts with increasing temperature. Figure 3b illustrates the temperature characteristics of this fiber Lyot-Sagnac interferometer; the transmission spectra shifted linearly as the temperature increased. As shown in Figure 3b, when the temperature changes between 40 and 41 °C, from the linear fit, the temperature sensitivity is −17.99 nm/°C for this fiber Lyot-Sagnac interferometer. To demonstrate the high temperature sensitivity characteristics of this fiber Lyot-Sagnac interferometer, we compared it with the conventional Sagnac interferometer. From Figure 3b, the Lyot-Sagnac interferometer has a much higher temperature sensitivity than that of the conventional one (1.46 nm/°C). The high temperature response is induced by the Vernier effect, which is an efficient method for the improvement of temperature sensitivity and enhancing measurement accuracy [13]; when the temperature changes, the transmission of the single Sagnac loop sensor will shift, and then the shift of Lyot-Sagnac envelope will be magnified by a certain factor. Because of its high temperature sensitivity, this interferometer can meet high temperature sensitivity requirements in various applications, especially in the fields that require high temperature measurement resolution.

**Figure 4:**
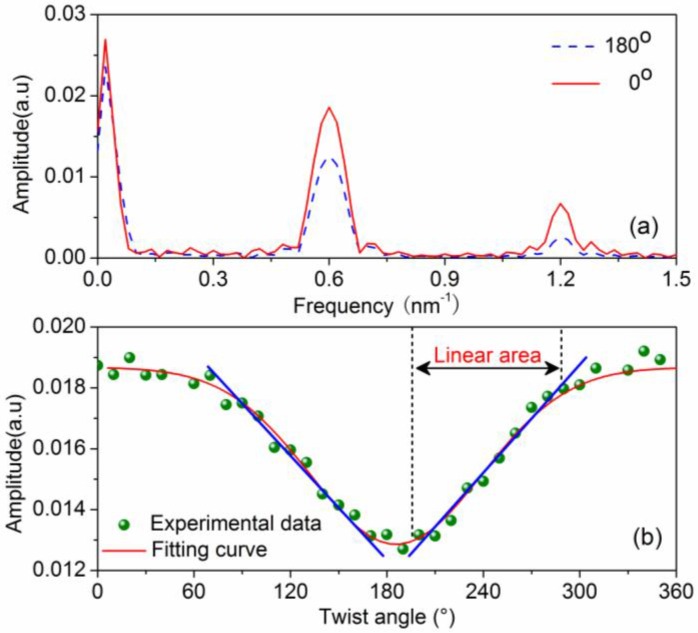
(**a**) Fast-Fourier transform (FFT) of the spectra; (**b**) Torsion responses of the Lyot-Sagnac interferometer.

To verify the torsion-sensing capability and torsion characteristics of the Lyot-Sagnac interferometer, we tested the torsion responses of the Lyot-Sagnac interferometer at room temperature. One end of the SMF in between the two sections of HiBi fiber was fixed firmly, and the other end was twisted by the torsion device. When torsion is applied to the SMF, the angle between the fast axes of the two sections of HiBi fiber will change, resulting in changes in the fringe visibility of the spectrum in the interferometer. Consequently, the fringe visibility changes with the applied torsion. Therefore, the applied torsion can be detected and evaluated by measuring the evolution of fringe visibility. In order to clearly observe the change in fringe visibility, we performed a fast Fourier-transform on the spectra of the interferometer to obtain the spatial frequency spectra. Figure 4a illustrates the spatial spectra of the interferometer versus twist angle variations at 0 and 180°. Figure 4b shows the response as a function of the twist angle. As shown in Figure 4b, the intensity and twist angle perfectly conform to Sine relationship. The experimental results are in good agreement with the theoretical analysis from Equation (4). 

**Figure 5:**
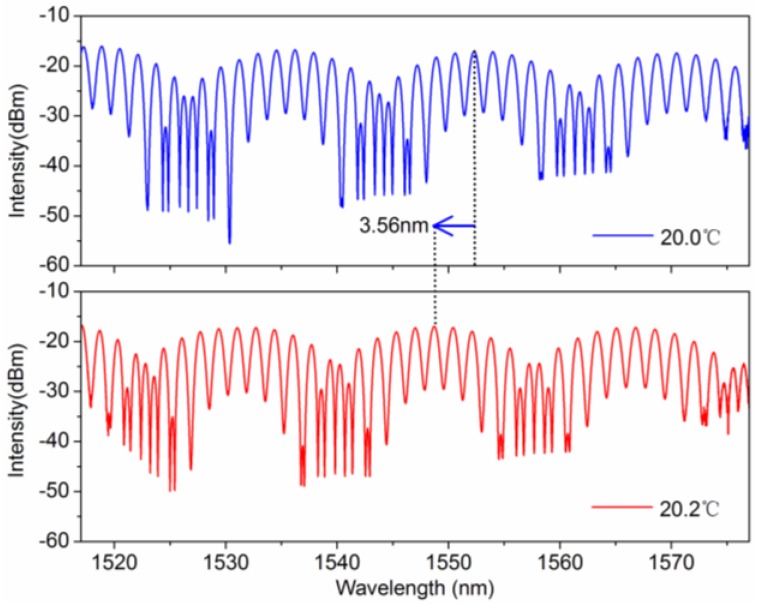
Spectra of Lyot-Sagnac sensor at the 20.0 °C and 20.2 °C.

**Figure 6:**
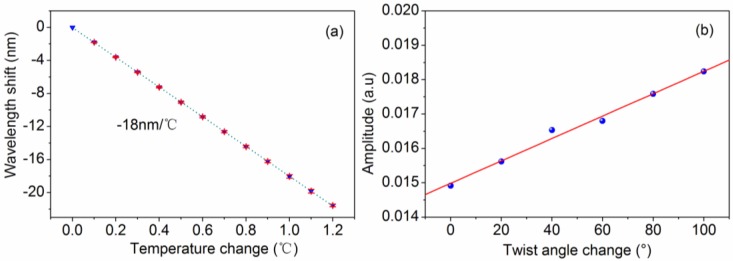
(**a**) Temperature responses of the Lyot-Sagnac interferometer under different twist angles; (**b**) Torsion responses of the Lyot-Sagnac interferometer-based sensor.

In practical applications, multi-parameter simultaneous measurement is an important subject for many fiber sensors; in order to understand the cross influence between temperature and torsion measurements, we investigated the performances of the Lyot-Sagnac interferometer-based sensor by introducing the alternative variation of temperature and torsion. Firstly, in order to study the effect of torsion on temperature characteristic, the temperature responses were measured under different twist angles. Figure 5 shows the transmission spectra of the Lyot-Sagnac interferometer at 20.0 and 20.2 °C when the twist angle is set at 100°. Figure 5 shows that, as the temperature increases, the spectrum showed blue shift and the total wavelength shift was up to 3.56 nm, and the fringe visibility was largely unchanged. Figure 6a presents the wavelength shift versus the temperature when the twist angle is maintained at 0°, 20°, 40°, 60°, 80°, and 100°. Under different twist angles, the temperature sensitivities are almost the same. From the linear fit, the temperature sensitivities are approximately −18 nm/°C. Figure 6b shows the torsion response of the Lyot-Sagnac sensor versus 100° twist angle variations. Within this twist angle range, there is a linear relation between the intensity and twist angle. The intensity increased linearly as the twist angle changed by 100°, and the measurement range can be seen as the linear area of the measurement range shown in Figure 4b.

## 4. Conclusions

We propose a Lyot-Sagnac interferometer by inserting two sections of HiBi fibers into the fiber loop and experimentally demonstrating its temperature and twist-sensing capability. The two inserted sections of HiBi fiber have different functions; while one section acts as the temperature-sensitive region, the other can be used as a reference fiber. The experimental results show that the proposed sensor has high sensitivity to temperature, and a 17.99 nm/°C temperature sensitivity is obtained. Furthermore, we calibrated and tested the twist responses of the proposed sensor, and the test results reveal that the fringe visibility and twist angle perfectly conform to Sine relationship. Therefore, the twist angle can be detected and evaluated by measuring the evolution of the fringe visibility. Because of its high temperature sensitivity, ease of fabrication, and low cost, this interferometer could find various applications in different areas. Further investigation will focus on the optimization of the structure parameters for different practical applications.
